# Mutation of Protoporphyrinogen IX Oxidase Gene Causes Spotted and Rolled Leaf and Its Overexpression Generates Herbicide Resistance in Rice

**DOI:** 10.3390/ijms23105781

**Published:** 2022-05-21

**Authors:** Xin Liu, Xiao-Jian Deng, Chun-Yan Li, Yong-Kang Xiao, Ke Zhao, Jia Guo, Xiao-Rong Yang, Hong-Shan Zhang, Cong-Ping Chen, Ya-Ting Luo, Yu-Lin Tang, Bin Yang, Chang-Hui Sun, Ping-Rong Wang

**Affiliations:** 1 State Key Laboratory of Crop Gene Exploration and Utilization in Southwest China, Sichuan Agricultural University, Chengdu 611130, China; liu130126@126.com (X.L.); sunhui0307@163.com (C.-H.S.); 2 Rice Research Institute, Sichuan Agricultural University, Chengdu 611130, China; lcy3208547178@163.com (C.-Y.L.); xiao13881891741@outlook.com (Y.-K.X.); zhaoke0607@163.com (K.Z.); guojia6@stu.sicau.edu.cn (J.G.); yangxr202011@163.com (X.-R.Y.); hszhang123@gmail.com (H.-S.Z.); cpchen2501@163.com (C.-P.C.); 15984509179@163.com (Y.-T.L.); tyl18892891705@163.com (Y.-L.T.); yb18080897532@163.com (B.Y.)

**Keywords:** rice (*Oryza sativa*), protoporphyrinogen IX oxidase, protoporphyrinogen IX, protoporphyrin IX, spotted leaf, rolled leaf, herbicide resistance, diethyl ether herbicide

## Abstract

Protoporphyrinogen IX (Protogen IX) oxidase (PPO) catalyzes the oxidation of Protogen IX to Proto IX. PPO is also the target site for diphenyl ether-type herbicides. In plants, there are two *PPO* encoding genes, *PPO1* and *PPO2*. To date, no *PPO* gene or mutant has been characterized in monocotyledonous plants. In this study, we isolated a *spotted and rolled leaf* (*sprl1*) mutant in rice (*Oryza sativa*). The spotted leaf phenotype was sensitive to high light intensity and low temperature, but the rolled leaf phenotype was insensitive. We confirmed that the *sprl1* phenotypes were caused by a single nucleotide substitution in the *OsPPO1* (*LOC_Os01g18320*) gene. This gene is constitutively expressed, and its encoded product is localized to the chloroplast. The *sprl1* mutant accumulated excess Proto(gen) IX and reactive oxygen species (ROS), resulting in necrotic lesions. The expressions of 26 genes associated with tetrapyrrole biosynthesis, photosynthesis, ROS accumulation, and rolled leaf were significantly altered in *sprl1*, demonstrating that these expression changes were coincident with the mutant phenotypes. Importantly, *OsPPO1*-overexpression transgenic plants were resistant to the herbicides oxyfluorfen and acifluorfen under field conditions, while having no distinct influence on plant growth and grain yield. These finding indicate that the *OsPPO1* gene has the potential to engineer herbicide resistance in rice.

## 1. Introduction

Tetrapyrroles play key roles in various biological processes, such as photosynthesis and respiration. Higher plants produce four classes of tetrapyrroles, including siroheme, heme, phytochromobilin, and chlorophyll (Chl). Tetrapyrroles are synthesized via a multibranched pathway. All four classes of tetrapyrroles share the six biosynthesis steps from glutamate to uroporphyrinogen III. The three subsequent steps from uroporphyrinogen III to protoporphyrin IX (Proto IX) are common to Chl, heme, and phytochromobilin synthesis. Finally, Fe^2+^ ion is inserted into Proto IX in the formation of heme and phytochromobilin, while Mg^2+^ ion is inserted into Proto IX in the formation of Chl [1]. So far, 33 genes for Chl and heme biosynthesis have been identified in *Arabidopsis* [1,2]. However, only 15 genes for Chl and heme biosynthesis have been identified in rice [3,4,5,6,7,8,9,10,11,12,13,14,15].

Protoporphyrinogen IX (Protogen IX) oxidase (PPO) catalyzes the oxidation of Protogen IX to form Proto IX, which is the last common step in Chl and heme biosynthesis. There are two genes encoding PPO proteins (PPO1 and PPO2) in *Arabidopsis*, tobacco (*Nicotiana tabacum*), spinach (*Spinacia oleracea*), and other plants, which share only 24% and 27% identity in *Arabidopsis* and tobacco, respectively [16,17,18,19]. Moreover, PPO1 is plastid-localized, and PPO2 is mitochondria-localized [16,17]. The *hemG* gene encoding PPO was first identified from *Escherichia coli* (*E. coli*) [20]. Up to now, *PPO* genes have been characterized from human, yeast, and plants [16,21,22]. In plants, *Arabidopsis*, tobacco, and spinach *PPO* genes have been isolated by the functional complementation of the *E. coli hemG* mutant [16,17,18]. In *Arabidopsis*, transgenic plants expressing the antisense *PPO1* gene showed the necrotic lesions phenotype, homozygous *ppo1* null mutants from T-DNA insertion displayed etiolated cotyledons and a seedling lethal, and heterozygous *ppo1-1/+* plants showed reproductive growth defects [23,24,25]. In tobacco, the *PPO1* antisense plants showed a reduced growth rate and necrotic leaf damage and generated more necrotic leaf lesions under low-light than under high-light exposure [26].

PPO is a molecular target site for photodynamically active herbicides of the diphenyl ether (DPE) type, which inhibits PPO activity by competing with its substrate Protogen IX. When DPE interacts with PPO, excess Protogen IX diffuses from chloroplasts into the cytoplasm and is oxidized to photosensitizing Proto IX by an unspecific plasma membrane-bound peroxidase. Proto(gen) IX absorbs excessive light energy, which finally results in the formation of highly reactive oxygen species (ROS). Then, ROS provoke the rapid degradation of membranes, proteins, and DNA and eventually lead to cell death [27,28]. The overexpression of *Bacillus subtilis* and *Arabidopsis PPO* genes in tobacco generated resistance to the DPE herbicides oxyfluorfen and acifluorfen, respectively [28,29]. Transgenic rice plants overexpressing *Bacillus subtilis*, *Arabidopsis*, human, and *Myxococcus xanthus* *PPO* genes were resistant to oxyfluorfen under controlled growth conditions [30,31,32,33,34,35].

Nonetheless, no *PPO* gene or mutant has been reported in monocotyledonous plants, and the function of *PPO* in rice remains to be explored. In the present study, we isolated a *spotted and rolled leaf* (*sprl1*) mutant from the ethyl methanesulfonate (EMS)-treated indica rice restorer line Lehui188 (188R). The *sprl1* mutant exhibited necrotic lesions on leaves throughout development and extremely inwardly rolled leaves before the three-leaf stage. We cloned the mutated gene and confirmed that the mutant phenotype of *sprl1* was caused by a single nucleotide substitution in *LOC_Os01g18320* gene encoding OsPPO1. The *sprl1* mutant accumulated excess Proto(gen) IX and ROS. Unlike the *PPO1* antisense tobacco plants, the *sprl1* leaves displayed more necrotic lesions under high light intensity or low temperature. Importantly, the overexpression of the *OsPPO1* gene generated resistance to the DPE herbicides oxyfluorfen and acifluorfen under field conditions, while having no detrimental effect on plant growth or grain yield, indicating the application potential of *OsPPO1* in rice.

## 2. Results

### 2.1. Phenotypic Characterization of the sprl1 Mutant

**Figure 1:**
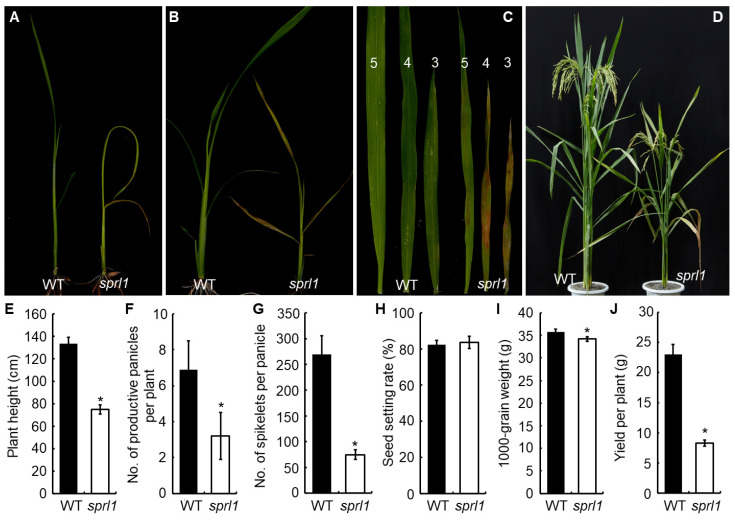
Comparison of phenotypes and major agronomic traits between the *sprl1* mutant and wild type (WT). (**A**) Plants at the two-leaf stage. (**B**) Plants at the four-leaf stage. (**C**) The top three leaves at the five-leaf stage; 5, 4, and 3 indicate the fifth, the fourth, and the third leaf from the bottom, respectively. (**D**) Plants at the grain filling stage. (**E**–**J**) Comparison of major agronomic traits between *sprl1* and WT. Error bars represent standard deviations of three independent biological replicates. The statistically significant differences were performed by Student’s *t* test. * *p* < 0.05.

The *sprl1* mutant displayed reddish-brown lesions on the leaves throughout the whole growth period (Figure 1A–D). Before the three-leaf stage, the mutant also showed extremely inwardly rolled leaves. In addition, the second and third leaf tip of *sprl1* did not protrude from its last leaf blades in time, resulting in the formation of an O-shape between the newly grown leaf blades and the previous one (Figure 1A and Figure S1A). After the four-leaf stage, the mutant leaves develop normally, and the reddish-brown spots were initiated from the tip of the leaf and then gradually spread to the whole leaf (Figure 1B). Nonetheless, the spots gradually reduced with the growth of *sprl1* plants, and only a few lesions were observed on the tip of the *sprl1* flag leaves after the heading stage (Figure 1C,D and Figure S1B,C).

The mutation of *sprl1* also affected the plant’s growth and agronomic traits. For instance, the mutant showed growth retardation, leading to a delay of the heading date by about 7 days compared with the wild type. At maturity, the plant height, the number of productive panicles per plant, the spikelet number of the main panicle, and the 1000-grain weight were significantly reduced in the *sprl1* mutant by 44.0%, 53.6%, 72.2%, and 4.5%, respectively, when compared to the wild type (Figure 1E–I). Consequently, the grain yield per plant was dramatically decreased by 63.9% in *sprl1* (Figure 1J).

**Figure 2:**
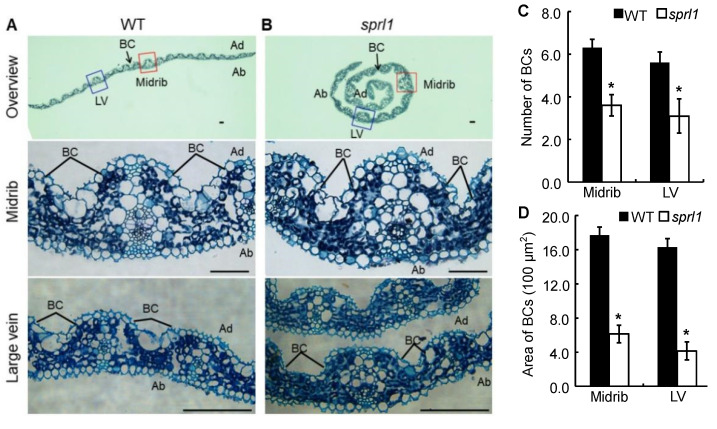
Paraffin sectioning analysis of leaf blades between the *sprl1* mutant and its wild type (WT). (**A**,**B**) Transverse section of the overview, midrib, and large vein at the middle of the second leaf at the two-leaf stage in WT and *sprl1*, respectively. (**C**,**D**) Statistical analysis of the number and area of bulliform cells, respectively. Ab, abaxial surface; Ad, adaxial surface; BC, bulliform cell; LV, large vein. Scale bar = 50 μm. Data are mean ± SD (n ≥ 6). Asterisks * indicate statistically significant differences compared with the wild type at *p* < 0.05.

To explore the cause of the rolled leaves at the seedling stage, we observed a cross-section structure of the second leaf in the wild type and *sprl1* mutant by paraffin sectioning at the two-leaf stage. As shown in Figure 2, the number and size of bulliform cells displayed distinct differences between the *sprl1* and the wild type leaves. More specifically, the bulliform cells were arranged in groups of five to seven cells in the wild type leaf blades, whereas they were arranged in groups of two to four cells in the *sprl1* leaf blades (Figure 2C). Consequently, the area of bulliform cells in *sprl1* was significantly decreased by 65% and 75% in the midrib and large vein, respectively (Figure 2D). These data suggest that the rolling leaf phenotype of *sprl1* results from the reduced number and size of bulliform cells in the leaf blades.

### 2.2. Physiological Characterization of the sprl1 Mutant

**Figure 3:**
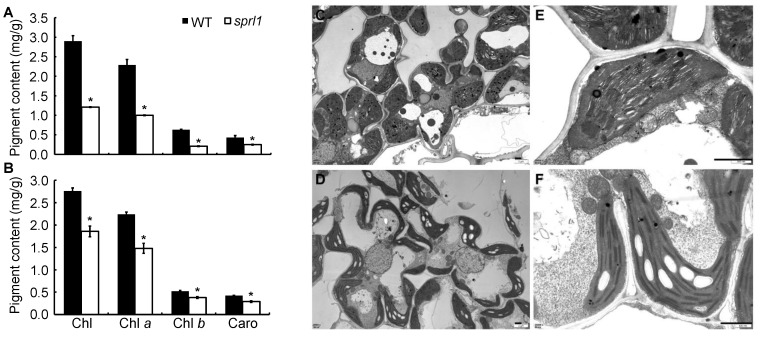
Photosynthetic characterization of the *sprl1* mutant. (**A**,**B**) Photosynthetic pigment contents in leaves of *sprl1* and WT at the seedling and heading stages, respectively (in mg g fresh weight^−1^). Data are the mean ± SD from three independent biological experiments. Asterisks * indicate statistically significant differences between WT and *sprl1* at *p* < 0.05 by Student’s *t* test. (**C**,**E**) Mesophyll cell and chloroplast of the wild type at the seedling stage, respectively. (**D**,**F**) Mesophyll cell and chloroplast of *sprl1* at the seedling stage, respectively. Chl, chlorophyll. Caro, carotenoids. Scale bars = 1 μm.

To investigate the photosynthetic characteristics of *sprl1*, we measured the photosynthetic pigment contents in the mutant and its wild type. Compared to the wild type, the contents of total Chl, Chl *a*, Chl *b*, and Caro in *sprl1* decreased by 58.3%, 56.3%, 66.7%, and 41.9% at the seedling stage and by 32.6%, 33.9%, 26.9%, and 31.0% at the heading stage (Figure 3A,B), respectively. We also characterized the ultrastructure of the chloroplasts in the leaves of *sprl1* and its wild type at the seedling stage using transmission electron microscopy. Compared to the wild type, the mutant chloroplasts were tiny and abnormal, and the grana stacks were thinner. Additionally, the number of starch granules in the *sprl1* chloroplast was obviously increased (Figure 3C–F). These results suggested that the mutation of *sprl1* reduced pigment contents and arrested chloroplast development.

### 2.3. ROS Accumulation in the sprl1 Mutant

**Figure 4:**
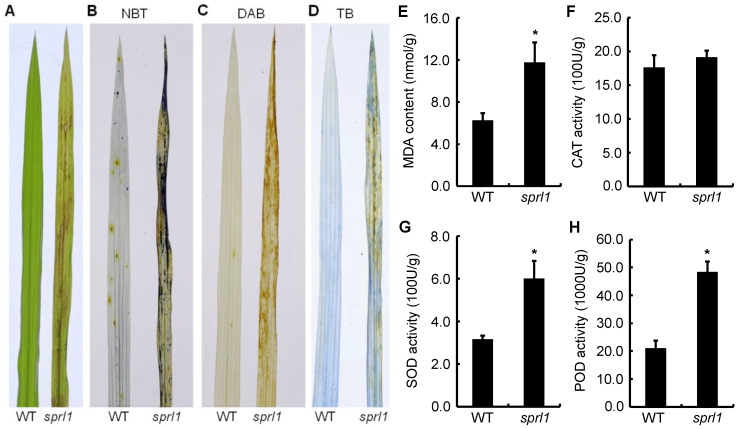
Detections of ROS accumulation and senescence-related indices in *sprl1* mutant at the five-leaf stage. (**A**) The fourth leaf blade at the five-leaf stages. (**B**) NBT staining. (**C**) DAB staining. (**D**) TB staining. (**E**) MDA content in nmol g fresh weight^−1^. (**F**) CAT activity in 100U g fresh weight^−1^. (**G**) SOD activity in 100U g fresh weight^−1^. (**H**) POD activity in 1000U g fresh weight^−1^. Data represent mean ± SD of three independent biological experiments. Asterisks * indicate statistically significant differences between WT and *sprl1* at *p* < 0.05 by Student’s *t* test.

The over-accumulation of ROS causes necrotic lesions on the leaves of some mutants [4,36]. To determine whether *sprl1* leaves have ROS over-accumulation and cell death, a matched section of leaves from the mutant and its wild type was collected and assayed by nitro blue tetrazolium (NBT), 3,3′-diaminobenzidin (DAB), and trypan blue (TB) staining. In NBT and DAB staining, the leaves with lesions were dyed deep blue and brownish in the mutant, respectively, but no staining was observed in the wild type leaves (Figure 4B,C), indicating that O^2−^ and H_2_O_2_ were highly accumulated in the *sprl1* leaves compared with the wild type leaves. In TB staining, blue spots were visible in the *sprl1* leaves, while no blue spots were found on the wild type leaves, suggesting higher levels of cell death in the *sprl1* leaves (Figure 4D).

Malondialdehyde (MDA) is a product of membrane lipid peroxidation and an indicator of free radical production and consequent tissue damage [36]. So, we examined the level of MDA in the *sprl1* mutant at the seedling stage. As shown in Figure 4E, the content of MDA was significantly higher in the *sprl1* leaves than that in the wild type leaves. Meanwhile, we also examined the levels of antioxidative enzyme activities in the leaves, including catalase (CAT), superoxide dismutase (SOD), and peroxidase (POD). The results showed that the activities of SOD and POD in the *sprl1* leaves were remarkably elevated by 89.8% and 129.5%, respectively, compared to those in the wild type (Figure 4G,H), while there was no significant difference in the CAT level between *sprl1* and the wild type (Figure 4F).

### 2.4. The sprl1 Locus Mapped to a Putative Gene Encoding Protogen IX Oxidase

For genetic analysis of the mutant phenotype, we crossed the *sprl1* mutant with its wild type parent 188R. The resulting F_1_ plants all displayed a normal leaf phenotype. The F_2_ populations exhibited the segregation of the wild type plants and the mutant plants at the seedling stage, with a ratio of 3:1 (Table S3, χ^2^ < χ^2^_0.05_ = 3.84, *p* > 0.05), suggesting that the spotted and rolled leaf phenotype of *sprl1* is controlled by a single recessive nuclear gene.

**Figure 5:**
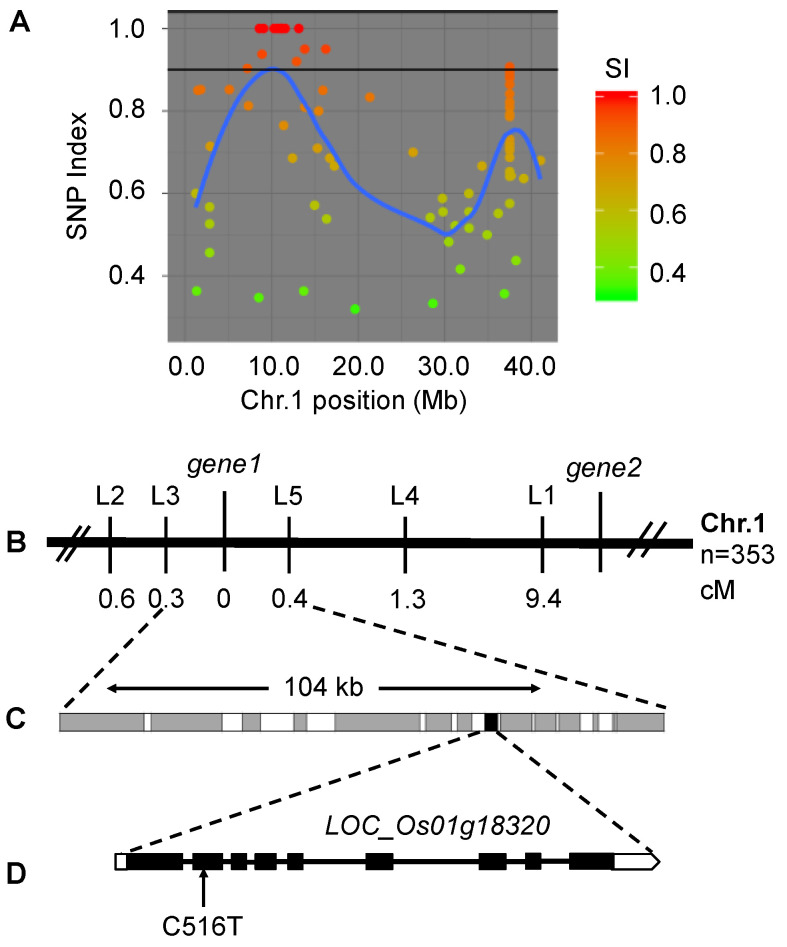
Gene mapping of the *sprl1* mutant. (**A**) SNP index plot for *sprl1* showed genetic linkage on chromosomes 1. (**B**) The *sprl1* locus was mapped to a 104 kb interval between InDel markers L3 and L5 using 353 recessive F_2_ individuals. (**C**) The 104 kb region contains 14 genes, and the candidate gene is *LOC_Os01g18320*. (**D**) *LOC_Os01g18320* is comprised of nine exons and eight introns, in which a single nucleotide C to T substitution occurred at the position of its coding region in the *sprl1* mutant.

To isolate the candidate gene of *sprl1*, we carried out whole-genome resequencing and a MutMap analysis. A bulked DNA pool from 30 mutant plants showing spotted and rolled leaves in the (*sprl1* × 188R) F_2_ population was sequenced and analyzed. Consequently, a linkage region was identified on the short arm of chromosome 1 (Figure 5A and Figure S2). In this region, two single nucleotide polymorphism (SNP) variations were found to be located in *LOC_Os01g18320* (*gene1*) and *LOC_Os01g22954* (*gene2*), whose SNP-index values were 1.0 and 0.92, respectively. Meanwhile, to further identify the *sprl1* mutant gene, we further analyzed 353 homozygous recessive individuals from the (*sprl1* × *japonica* cultivar Zhonghua 11) F_2_ mapping population, using five insertion/deletion (InDel) markers within the vicinity of the two gene loci (Table S4). Finally, only *gene1* locus fell into the 104 kb region between the InDel markers L3 and L5, at genetic distances of 0.3 cM and 0.4 cM, respectively (Figure 5B,C).

A MutMap analysis revealed that a missense variant occurred at an exon of the *LOC_Os01g18320* gene encoding Protogen oxidase 1 (PPO1) in tetrapyrroles biosynthesis. We amplified and sequenced *LOC_Os01g18320* in the *sprl1* mutant and its wild type, respectively. The result confirmed that *LOC_Os01g18320* carries a single nucleotide C-to-T substitution at position 516 in the *sprl1* mutant, consistent with the MutMap analysis. cDNA sequencing analysis showed that the C-to-T substitution occurred at position 427 in the cDNA of this gene (Figure 5D), causing only the amino acid Arg-143 to Trp change in the encoded protein. Therefore, *LOC_Os01g18320* was identified as the candidate gene of *sprl1* and designated as *OsPPO1*.

A sequence comparison showed that *OsPPO1* consists of nine exons and eight introns, with the full length genomic and cDNA sequences being 3246 bp and 1611 bp, respectively. *OsPPO1* encodes a 536-amino acid protein with a molecular mass of approximately 56.7 kD. Using the deduced amino acid sequence of OsPPO1, a BlastP search was conducted against the rice genome (http://rapdb.dna.affrc.go.jp/tools/blast, accessed on 2 December 2021). The BlastP results showed that there is one homologous gene (*LOC_Os04g41260*) of *OsPPO1* in rice, which is predicted to encode OsPPO2. OsPPO2 shares 27% identity with OsPPO1. While the OsPPO2 protein is predicted to be targeted to the mitochondria, the OsPPO1 protein contains a chloroplast-targeting sequence of 36 amino acid residues at its N terminus (http://www.cbs.dtu.dk/services/ChloroP, accessed on 2 December 2021, Figure S3A). A multiple amino acid sequence alignment indicated that the OsPPO1 protein has a high similarity to the PPO1 proteins from maize (*Zea mays*), barley (*Hordeum vulgare*), *Arabidopsis*, potato (*Solanum tuberosum*), and tobacco, with identities of 89, 88, 71, 70 and 69% respectively, but it only has low identities to the PPOs of other organisms such as *Bacillus subtilis*, *Homo sapiens*, and *Myxococcus xanthus* (24, 23, and 19% respectively). A phylogenetic analysis showed that OsPPO1 is more closely related to the homologs from monocotyledon maize and barley than to those from other species (Figure S3B). In addition, the sequence of OsPPO1 was also found to contain the Gly-rich motif GxGxxG as a dinucleotide binding site of many flavin-containing proteins (Figure S3A) [17,18,37].

### 2.5. Excessive Accumulation of Proto IX in sprl1 Mutant

**Figure 6:**
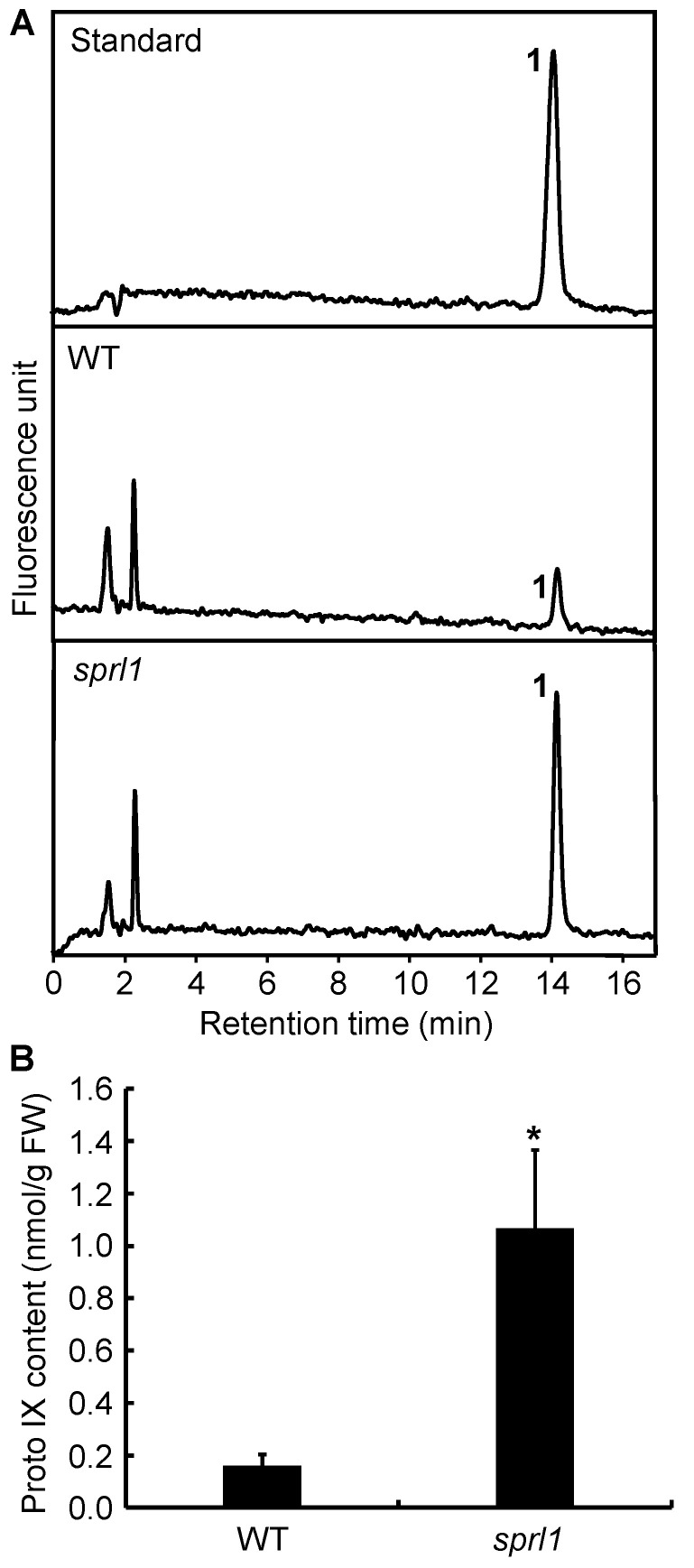
HPLC analysis of Proto IX. (**A**) The elution profiles of the Proto IX standard, WT, and *sprl1* mutant in leaves at the seedling stage, respectively. Peak 1, Proto IX. Elution profiles were detected by fluorescence, with excitation at 405 nm and emission at 625 nm. (**B**) Proto IX contents of WT and *sprl1* were quantified by using the Proto IX standard. Data are mean ± SD of three independent biological experiments. Asterisks indicate statistically significant differences between the WT and *sprl1* mutant at * *p* < 0.05 by Student’s *t* test.

Because Protogen IX is the substrate for PPO1 in tetrapyrroles biosynthesis, there should be an excessive accumulation of Protogen IX and photo-oxidized Proto IX when the plants have a defect in the PPO1 protein [26]. For a quantitative HPLC analysis, Protogen IX was first oxidized to fluorescent Proto IX. Then, the total content of Proto IX in the *sprl1* seedling was examined by HPLC according to the described method [26]. The results showed that the level of Proto IX in *sprl1* was significantly higher than that in the wild type. The content of Proto IX in *sprl1* was 1.07 nmol g^−1^ FW, but that in the wild type was only 0.16 nmol g^−1^ FW (Figure 6). Therefore, *sprl1* accumulated excess Proto(gen) IX, confirming that the mutant indeed has a defective PPO1 protein.

### 2.6. OsPPO1 Gene Rescued the Mutant Phenotype of sprl1

**Figure 7:**
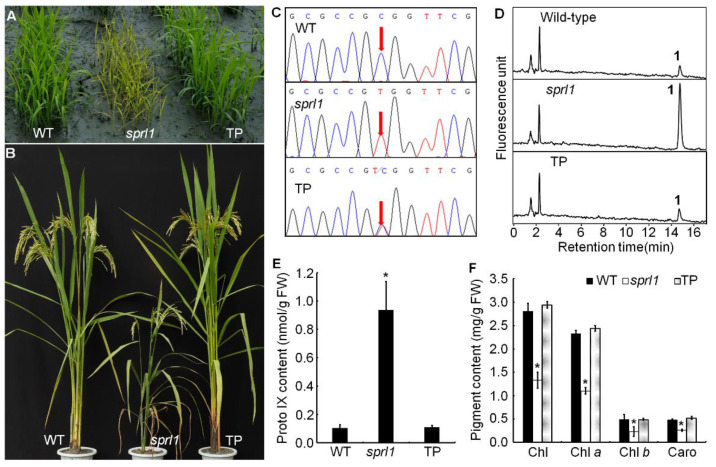
Functional complementation of *OsPPO1* in the *sprl1* mutant. (**A**,**B**) Phenotypes of WT, *sprl1*, and transgenic plants (TP) at the seedling and grain filling stage, respectively. (**C**) Identification of transgenic plants by sequencing. (**D**) The elution profiles of WT, *sprl1*, and TP at the seedling stage, respectively. Elution profiles were detected by fluorescence, with excitation at 405 nm and emission at 625 nm. Peak 1, Proto IX. (**E**) Proto IX contents of WT, *sprl1*, and TP at the seedling stage. (**F**) Pigment contents of WT, *sprl1*, and TP at the seedling stage. Data are mean ± SD of three independent biological experiments. Asterisks * indicate statistically significant differences between WT and *sprl1*, and between TP and *sprl1*, at *p* < 0.05 by Student’s *t* test.

To further confirm that the mutant phenotype of *sprl1* was caused by the mutation of the *OsPPO1* gene, we performed a complementation analysis. The construct pCAMBIA1300-*OsPPO1*, containing a 5528 bp *OsPPO1* genomic DNA fragment consisting of a 1949 bp upstream sequence, the entire coding region, and a 333 bp downstream sequence, was introduced into the *sprl1* mutant by *Agrobacterium*-mediated transformation. As a result, 18 independent transgenic lines were successfully obtained. Among them, 11 lines were identified as positive transgenic lines by the PCR examination and sequencing analysis, which displayed normal phenotypes compared to the wild type, including the leaf blade, plant height, and other major agronomic traits (Figure 7A–C and Figure S4). Furthermore, the contents of Proto IX and photosynthetic pigments were recovered to the wild type levels (Figure 7D–F). Overall, these results confirmed that the single nucleotide mutation in *OsPPO1* (*LOC_Os01g18320*) is responsible for the spotted and rolled leaf phenotype of *sprl1*.

### 2.7. OsPPO1 Gets Localized to Chloroplast

**Figure 8:**
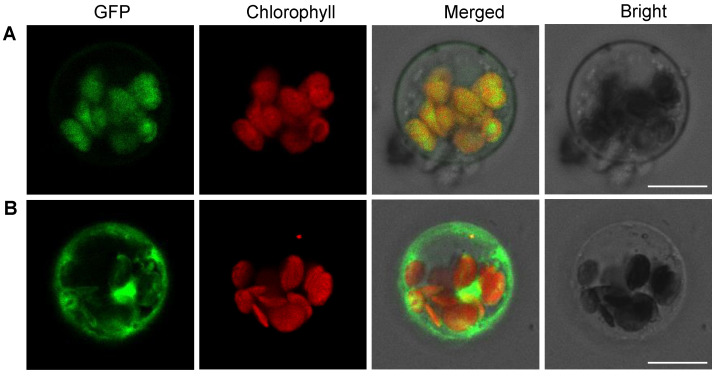
Subcellular localization of the OsPPO1 protein. (**A**) GFP signals of the OsPPO1-GFP fusion protein. (**B**) GFP signals of the empty vector. Fluorescence signals were visualized using a laser-scanning confocal microscope. Green fluorescence displays the GFP, red fluorescence shows chloroplast autofluorescence, yellow fluorescence indicates images with the two types of fluorescence merged, and bright-field images show rice protoplasts. Scale bars = 10 μm.

OsPPO1 was predicted to contain a chloroplast transit peptide with 36 amino acid residues at its N-terminus by TargetP and ChloroP [38]. To determine the subcellular localization of the OsPPO1 protein in vivo, we created an expression vector with GFP translationally fused to the OsPPO1 protein under the control of the CaMV 35S promoter. Subsequently, transient expressions of p35S:*OsPPO1*-*GFP* and p35S:*GFP* (as control) in rice protoplasts were observed using laser scanning confocal microscopy. As shown in Figure 8A, the green fluorescent signals of the p35S:OsPPO1-GFP fusion protein were clearly co-localized with the red autofluorescence of chlorophyll in the chloroplasts, but the green fluorescence of p35S:GFP could be seen in the whole cell (Figure 8B). The results confirmed that OsPPO1 is targeted to the chloroplast.

### 2.8. Light and Temperature Sensitivity of sprl1 Mutant

**Figure 9:**
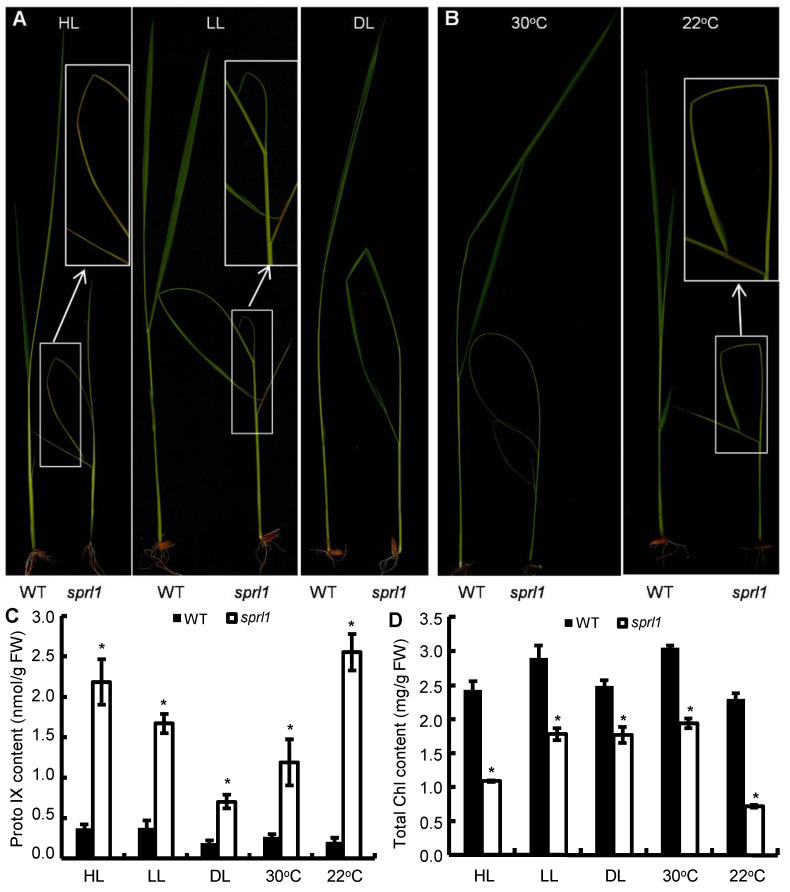
Phenotypic comparison of the *sprl1* mutant and its wild type (WT) grown in the growth chamber under various light intensity and temperature conditions. (**A**) 12-day-old seedlings grown under 12 h of high light (HL, 300 μmol m^−2^ s^−1^), low light (LL, 80 μmol m^−2^ s^−1^), and dim light (DL, 20 μmol m^−2^ s^−1^) conditions at 28 °C/12 h of darkness at 26 °C, respectively. (**B**) 12-day-old seedlings grown under constant 30 °C and 22 °C conditions, respectively, under 12 h of low light (LL, 80 μmol m^−2^ s^−1^)/12 h of darkness. (**C**,**D**) Contents of Proto IX and Chl, respectively, in WT and *sprl1* under various light intensity and temperature conditions. Data are mean ± SD of three independent biological experiments. Asterisks * indicate statistically significant differences between WT and *sprl1* at *p* < 0.05 by Student’s *t* test.

Transgenic tobacco plants expressing *PPO1* antisense RNA displayed more necrotic lesions on their leaves under low- compared to under high-light conditions [26]. To investigate whether the *sprl1* mutant has a similar phenotype as the antisense tobacco plants, we treated the *sprl1* mutant and its wild type using various light intensities, including high light (HL, 300 μmol m^−2^ s^−1^), low light (LL, 80 μmol m^−2^ s^−1^), and dim light (DL, 20 μmol m^−2^ s^−1^). As a result, the *sprl1* mutant displayed more necrotic lesions under HL conditions than under LL conditions, and no obvious necrotic lesion was observed under DL conditions (Figure 9A and Figure S5A–C), which was a different light sensitivity than the *PPO1* antisense tobacco plants. HPLC analysis and photosynthetic pigment determination showed that the *sprl1* mutant had remarkably higher accumulation of Proto IX and lower contents of pigments than its wild type (Figure 9C,D and Figure S5F–H), but the differences in the Proto IX levels and pigment contents between *sprl1* and the wild type were obviously reduced from HL to LL to DL, which was consistent with the corresponding phenotypes of the necrotic lesion under different light intensities.

On the other hand, we also treated *sprl1* and its wild type using two different temperatures (22 °C and 30 °C) under LL conditions, respectively. Consequently, the *sprl1* mutant showed severe necrotic lesions under 22 °C conditions, but only a few necrotic lesions were observed under 30 °C conditions (Figure 9B and Figure S5D,E). Correspondingly, though *sprl1* had remarkably higher Proto IX levels and lower pigment contents than the wild type (Figure 9C,D and Figure S5F–H), the differences in the Proto IX and pigment contents between *sprl1* and the wild type under 30 °C were obviously smaller than those under 22 °C.

In addition, the 12-day-old seedlings of *sprl1* all displayed seriously rolled leaves under three light intensities and two temperature conditions (Figure 9A,B and Figure S5A–E), indicating that the rolled leaf phenotype of *sprl1* was different from its spotted phenotype in terms of its sensitivity to light intensity and temperature conditions.

Therefore, the spotted leaf phenotype of the *sprl1* mutant is sensitive to light intensity and temperature, but the rolled leaf phenotype of *sprl1* is insensitive.

### 2.9. Expression Pattern Analysis of OsPPO1

**Figure 10:**
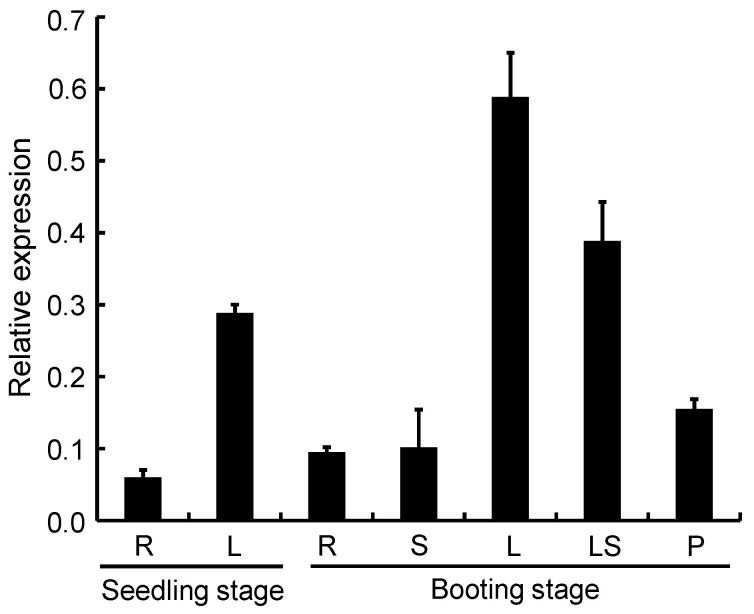
Expression analysis of the *OsPPO1* gene by qRT-PCR. The expression levels in the root (R), leaf (L), stem (S), leaf sheath (LS), and young panicle (P) of the wild type were determined at the seedling and booting stages. The transcript levels of *OsPPO1* were normalized using the rice *Actin 1* gene as an internal control. Data are mean ± SD of three independent biological replicates.

To investigate the expression pattern of the *OsPPO1* gene, we examined *OsPPO1* transcription levels in different organs at different development stages by qRT-PCR. As shown in Figure 10, the *OsPPO1* transcript was present in all the organs tested: the root and leaf blade at the seedling stage, and the root, stem, leaf blade, leaf sheath, and young panicle at the booting stage. Among them, the leaf blade at the booting stage had the strongest expression, followed by the leaf sheath at the booting stage and the leaf blade at the seedling stage. The root, stem, and young panicle had lower expression levels.

### 2.10. Expression Analysis of the Genes Associated with Tetrapyrrole Synthesis, Photosynthesis, ROS Accumulation, and Rolled Leaf

**Figure 11:**
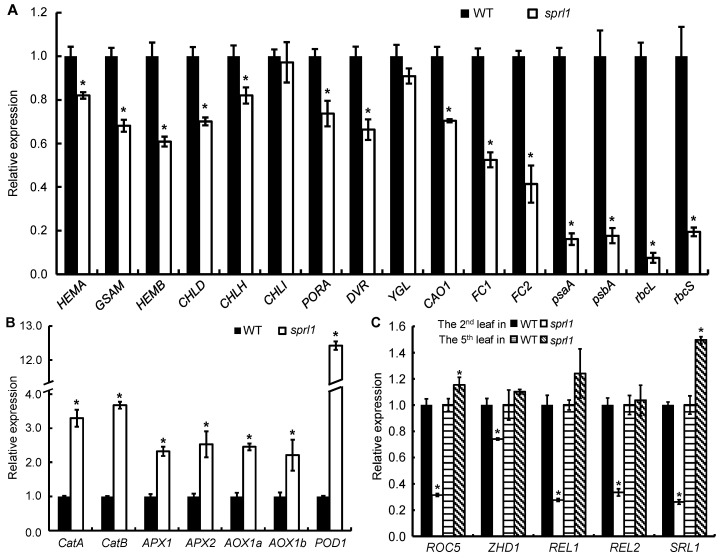
Expression analyses of genes in the *sprl1* mutant and its wild type (WT). (**A**) Expression levels of the genes associated with tetrapyrrole biosynthesis and photosynthesis at the seedling stage. (**B**) Expression levels of the genes associated with ROS accumulation at the seedling stage. (**C**) Expression levels of the genes associated with rolled leaf in the second leaf blade at the two-leaf stage (Figure 1A) and the fifth leaf blade at the five-leaf stage. Expression levels were normalized using the rice *Actin 1* gene as an internal reference. The expression level of each gene in WT was set to 1.0, and those in *sprl1* were calculated accordingly. Data are mean ± SD of three independent biological replicates. Asterisks indicate statistically significant differences between WT and *sprl1* at * *p* < 0.05 (with Student’s *t* test).

Since *sprl1* accumulated excess Proto(gen) IX and ROS, we examined the transcript levels of the 23 related genes at the seedling stage. Among them, 12 genes are involved in tetrapyrrole biosynthesis, including *HEMA* (glutamyl-tRNA reductase), *GSAM*, *HEMB* (5-aminolevulinate dehydratase), *CHLD*, *CHLH*, *CHLI*, *PORA*, *DVR*, *YGL*, *CAO1*, *FC1*, and *FC2* (ferrochelatase) [3,5,6,8,9,10,11,12,39,40]. Four genes are photosynthesis-related genes, including *psaA* and *psbA* (two reaction center polypeptides) and *rbcL* and *rbcS* (*Rubisco large subunit* and *small subunit*) [41]. Seven genes are involved in active oxygen scavenging, including *catA* and *catB* (catalase), *APX1* and *APX2* (ascorbate peroxidase), *AOX1a* and *AOX1b* (alternative oxidase), and *POD1* (peroxidase) [12,42,43,44,45]. As shown in Figure 11A, except for *OsCHLI* and *YGL*, the expression levels of the genes associated with tetrapyrrole synthesis and photosynthesis were significantly decreased, but the expressions of the genes associated with ROS accumulation were all dramatically increased in the *sprl1* mutant (Figure 11B). These data are consistent with the pleiotropic mutant phenotypes of *sprl1*, such as the reduced contents of photosynthetic pigments, the arrested development of chloroplast, the excessive accumulation of Proto(gen) IX and ROS, and necrotic leaves.

Because *sprl1* displayed a leaf rolling phenotype before the three-leaf stage, we also assayed the transcript levels of leaf rolling-related genes in the second leaf blade at the two-leaf stage and the fifth leaf blade at the five-leaf stage, respectively, including *ROC5* (*Rice Outermost Cell-specific gene5*), *ZHD1* (zinc finger homeodomain class homeobox transcription factors), *REL1* (*Rolled and Erect Leaf 1*), *REL2* (*Rolled and Erect Leaf 2*), and *SRL1* (*SEMI-ROLLED LEAF1*) [46,47,48,49,50]. The five genes were all remarkably down-regulated in the second leaf blade, but the expression levels of *ZHD1*, *REL1*, and *REL2* returned to the wild type levels, even as *ROC5* and *SRL1* were significantly up-regulated in the fifth leaf blade (Figure 11C). This result was highly consistent with the rolled leaf phenotype of *sprl1*.

### 2.11. Overexpression Analyses of OsPPO1

**Figure 12:**
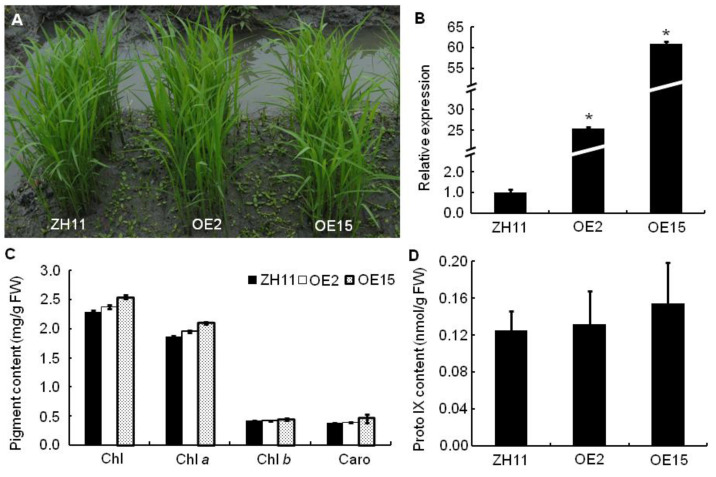
Phenotypic and physiological characterization of the control cultivar ZH11 and the overexpressing lines at the seedling stage. (**A**) Plants at the seedling stage. (**B**) Relative expression levels of the *OsPPO1* gene. The expression level in ZH11 was set to 1.0, and those in the overexpressing lines were calculated accordingly. (**C**) Photosynthetic pigments contents. (**D**) Proto IX contents. OE2 and OE15, transgenic lines overexpressing *OsPPO1*. Data are mean ± SD of three independent biological experiments. Asterisks indicate statistically significant differences between ZH11 and the overexpressing lines at * *p* < 0.01 (with Student’s *t* test).

To further characterize the function of *OsPPO1*, we phenotyped rice plants overexpressing the *OsPPO1* gene. The overexpression vector pCAMBIA2300-*OsPPO1*, containing the full-length cDNA sequence of the *OsPPO1* gene under the control of the CaMV 35S promoter, was constructed and then transformed into *japonica* cultivar Zhonghua 11 (ZH11). Consequently, 16 positive transgenic lines were obtained, in which 7 lines showed an extremely significant increase in the expression level of *OsPPO1* compared with the control ZH11 (Figure S6). Morphologically, the *OsPPO1* overexpressing (OE) lines were indistinguishable from the ZH11 control plants, both with a normal leaf development and growth rate (Figure 12A,B, and Figure S7). Among them, the OE2 and OE15 lines had the highest expression of *OsPPO1*, which were about 25- and 60-fold higher than ZH11, respectively. Therefore, the OE2 and OE15 lines were chosen for further studies.

We investigated the photosynthetic pigment and Proto IX contents and major agronomic traits of the homozygous OE2 and OE15 lines. The contents of Chls, Caro, and Proto IX had no significant difference among the OE2, OE15, and ZH11 lines (Figure 12C,D). Moreover, their major agronomic traits, including the plant height, the number of productive panicles per plant, the spikelet number per panicle, the seed setting rate, the 1000-grain weight, and the yield per plant, had no significant change (Figure S7). Therefore, the overexpression of *OsPPO1* had no significant effect on the tetrapyrrole biosynthesis, photosynthetic pigment contents, major agronomic traits, or grain yield.

**Figure 13:**
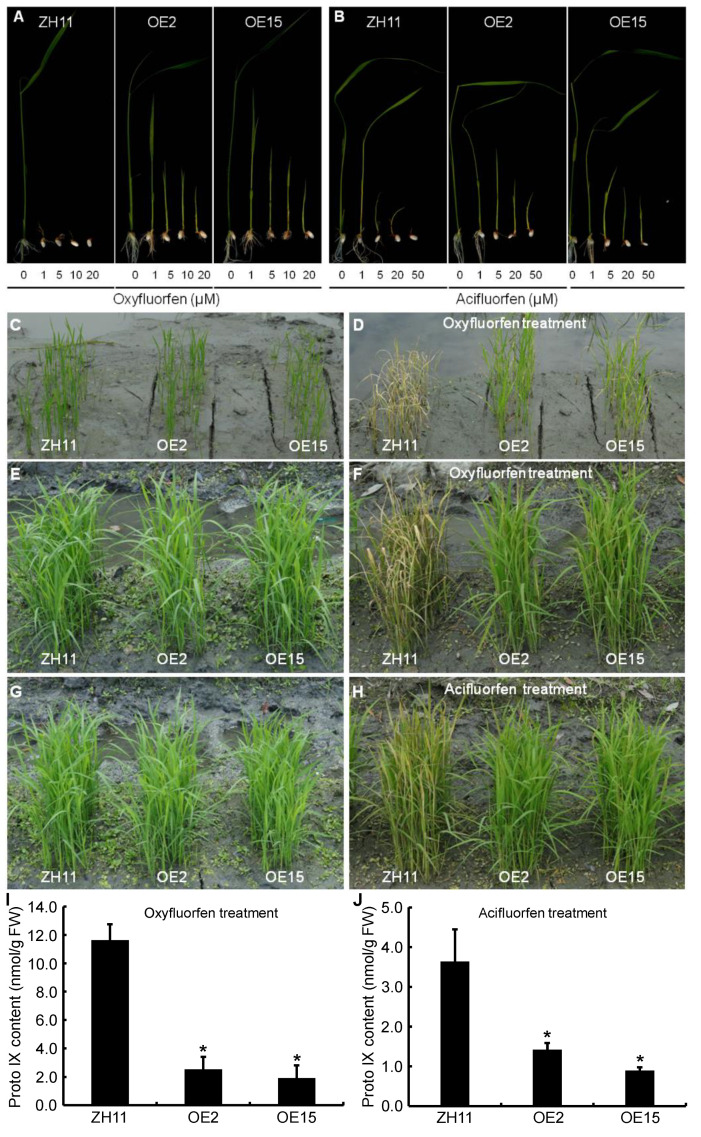
Herbicidal test for oxyfluorfen and acifluorfen resistances. (**A**,**B**) Seed germination test. Surface sterilized seeds were sown on a half-strength MS solid medium containing various concentrations of oxyfluorfen and acifluorfen, respectively. Photographs were taken 10 d after sowing. (**C**–**H**) Seedling growth test. (**C**,**D**) Phenotypes of the seedlings before oxyfluorfen treatment (2 weeks after sowing) and after 4 d of 125 μM oxyfluorfen treatment, respectively. (**E**,**F**) Phenotypes of the seedlings before oxyfluorfen treatment (3 weeks after sowing) and after 4 d of 125 μM oxyfluorfen treatment, respectively. (**G**,**H**) Phenotypes of the seedlings before acifluorfen treatment (3 weeks after sowing) and after 4 d of 600 μM acifluorfen treatment, respectively. (**I**,**J**) Accumulation of Proto IX in the 3-week-old seedlings after 24 h of 125 μM oxyfluorfen and 600 μM of acifluorfen treatment, respectively. Error bars represent standard errors of three independent biological experiments. The asterisk indicates a statistically significant difference compared with the control ZH11 at * *p* < 0.05.

In addition, the homozygous OE2 and OE15 lines were used for the oxyfluorfen resistance experiments. In the seed germination assay, the OE2, OE15, and ZH11 seeds were sowed on half-strength MS solid media supplemented with different concentrations of oxyfluorfen, respectively. As shown in Figure 13A, the ZH11 seeds did not germinate at 1 μM oxyfluorfen, but the OE2 and OE15 seeds could germinate and grow. When the concentration of oxyfluorfen gradually increased up to 20 μM, the transgenic seeds could still germinate, but the development of their shoots and roots was obviously retarded compared with the untreated transgenic seeds, and a few necrotic lesions could be found on their leaves. In the seedling growth experiment, two- or three-week-old rice seedlings were sprayed with 125 μM oxyfluorfen in a paddy field. After 4 d of oxyfluorfen treatment, almost all of the seedlings of the control ZH11 showed extremely severe necrotic lesions and eventually died. In contrast, the OE2 and OE15 seedlings had few necrotic lesions and could develop relatively normally (Figure 13C–F). The newly emerging leaves of OE2 and OE15 had no necrotic lesion. At maturity, the oxyfluorfen treated OE plants were not obviously different when compared to the untreated control ZH11 for major agronomic traits and grain yield (Figure S8). 

The homozygous OE2 and OE15 lines were also used for acifluorfen resistance experiments. Compared with the oxyfluorfen treatment, the seed germination and seedling growth of the OE lines and the control ZH11 were less sensitive to acifluorfen. The ZH11 seeds did not germinate at 50 μM acifluorfen, but the OE2 and OE15 seeds could germinate and grow (Figure 13B). When the three-week-old rice seedlings were sprayed with 600 μM acifluorfen, most of the ZH11 seedlings showed severe necrotic lesions after 4 d of acifluorfen treatment, but the OE2 and OE15 seedlings had only a few necrotic lesions (Figure 13G,H).

Third, we measured the Proto IX contents of the rice seedlings 24 h after the herbicide treatments. After 125 μM oxyfluorfen treatment, the Proto IX content in the control ZH11 reached 11.62 nmol g^−1^ FW, but those in OE2 and OE15 were only 2.51 and 1.91 nmol g^−1^ FW, respectively (Figure 13I). Similarly, after 600 μM acifluorfen treatment, the Proto IX content in the control ZH11 was 3.63 nmol g^−1^ FW, but those in OE2 and OE15 were 1.42 and 0.90 nmol g^−1^ FW, respectively (Figure 13J). These results indicated that the resistance of *OsPPO1* overexpression plants to herbicides should be attributed to them sustaining less accumulation of Proto IX after oxyfluorfen or acifluorfen treatment.

## 3. Discussion

To date, *PPO* genes have been characterized from *E. coli*, human, yeast, and plants [16,21,22]. In plants, *PPO* genes have been isolated by the functional complementation of the *E. coli hemG* mutant in *Arabidopsis*, tobacco, and spinach [16,17,18]. However, no *PPO* gene has been characterized in monocotyledonous plants. In this study, we isolated a *spotted and rolled leaf* (*sprl1*) mutant in rice and identified the causal *OsPPO1* (*LOC_Os01g18320*) gene by a MutMap cloning approach and functional complementation. In the *sprl1* mutant, a C-to-T substitution occurred in *OsPPO1*, resulting in an amino acid change in its encoded protein. The HPLC analysis showed that excess Proto(gen) IX was accumulated in *sprl1*. Moreover, the *sprl1* mutant phenotype can be rescued by complementation with the wild type *OsPPO1* gene. Therefore, we successfully identified the *PPO1* gene in rice and confirmed that a single nucleotide substitution in this gene caused the spotted and rolled leaf phenotype of *sprl1*.

*Arabidopsis* plants expressing the antisense *AtPPO1* gene showed a lesion-mimic phenotype, and tobacco plants expressing the antisense *NtPPO1* mRNA displayed necrotic leaf damage and a reduced growth rate [23,26]. In our study, the *sprl1* mutant also displayed a lesion mimic phenotype throughout the growth period (Figure 1 and Figure S1). The mutation of *OsPPO1* in *sprl1* caused the excess accumulation of photoreactive Proto(gen) IX, resulting in the over-accumulation of ROS, an increased content of MDA, and, eventually, cell death. However, unlike the above mutants of *Arabidopsis* and tobacco, the *sprl1* mutant displayed extremely inwardly rolled leaves at the seedling stage, which resulted from the decrease in the number and size of the bulliform cells in its leaf blades (Figure 1A, Figure 2 and Figure S1A). These data suggest that the *OsPPO1* gene not only participates in tetrapyrrole bioynthesis but also influences the development of the bulliform cells in leaf blades at the seedling stage, implying that *OsPPO1* could have new functions distinct from the *Arabidopsis* and tobacco *PPO1* genes.

Accumulating light-absorbing Proto(gen) IX evokes the generation of ROS and peroxidized lipids. However, the transgenic tobacco plants expressing the antisense *NtPPO1* gene could scarcely be distinguished from the wild type under high light (HL) conditions, but they grew more slowly and displayed more necrotic lesions under low light (LL) conditions [26]. In contrast, the rice *sprl1* mutant had more necrotic lesions under HL conditions but had only a few necrotic lesions under LL conditions and had no necrotic lesion under dim light (DL) conditions. Among these plants, HL-grown *sprl1* plants had the highest content of Proto IX and the lowest contents of photosynthetic pigments (Figure 9C,D and Figure S5F–H). Similarly, the *sprl1* mutant showed more necrotic spots and contained a higher content of Proto IX and lower levels of photosynthetic pigments under 22 °C than under 30 °C conditions (Figure 9C,D and Figure S5F–H). These data suggest that the mutated OsPPO1 protein in the *sprl1* mutant maintained partly catalytic activity for chlorophyll biosynthesis, but its catalytic activity was lower under HL and low temperature conditions than under LL and high temperature conditions. On the other hand, the *sprl1* plants still contained high levels of Proto(gen) IX under LL, DL, and high temperature conditions (Figure 9C), indicating that the Proto(gen) IX in *sprl1* was not converted into photosensitive Proto(gen) IX, so insufficient levels of photosensitive Proto(gen) IX could induce cellular damage under these conditions. Collectively, the photodynamic damage induced by Proto(gen) IX positively correlates with light intensity but negatively correlates with temperature in rice.

To date, several strategies have been employed to generate DEP-resistant transgenic rice by the heterologous overexpression of *PPO* genes. For example, transgenic rice expressing *Bacillus subtilis PPO* was found to be resistant to oxyfluorfen with respect to ion leakage, Chl loss, and lipid peroxidation. While most of these transgenic lines appeared to be normal, morphologically, their seed production varied [30,31]. In addition, rice plants overexpressing *Arabidopsis PPO1* or human *PPO* could germinate in the presence of 20 µM oxyfluorfen, whereas the wild type rice seeds could not [32,33]. Although showing some resistance to oxyfluorfen, transgenic rice plants expressing the human *PPO* gene showed growth retardation and the light-dependent formation of necrotic lesions [27,33]. Unlike previous studies, the transgenic rice plants overexpressing *OsPPO1* displayed no phenotypic differences when compared to wild type ZH11 plants, including in terms of photosynthetic pigment contents, plant growth, grain yield, etc. (Figure 12 and Figure S7). While the OE (OE2 and OE15) transgenic seeds showed no gross morphological difference under standard growth conditions, they showed resistance to oxyfluorfen in a variety of assays. Importantly, when two- or three-week-old seedlings were sprayed with 125 μM oxyfluorfen under field conditions, almost all of the control seedlings of ZH11 showed extremely severe necrotic lesions and eventually died, whereas the OE seedlings could develop relatively normally. In contrast with the transgenic rice overexpressing the heterologous *PPO* gene cited above, the over-expression *OsPPO1* transgenic lines seem to be highly resistant to oxyfluorfen, and they had no distinct influence on plant growth and grain yield. These results are likely attributed to the use of endogenous rice *OsPPO1* in our overexpression assays. Therefore, the *OsPPO* gene could have potential value in the engineering of DEP-herbicide resistance in rice.

## 4. Materials and Methods

### 4.1. Plant Materials

The *spotted and rolled leaf* (*sprl1*) mutant was isolated from the indica rice restorer line Lehui188 (188R) by ethyl methanesulfonate (EMS) mutagenesis. The rice materials were grown during the natural rice growing season in the experimental farms of Sichuan Agricultural University in Wenjiang District, Chengdu city, China [9]. For light intensity treatments, the rice seedlings were grown in the growth chamber under 12 h of high light (HL, 300 µmol m^−2^ s^−1^), low light (LL, 80 µmol m^−2^ s^−1^), and dim light (DL, 20 µmol m^−2^ s^−1^) at 28 °C/12 h of dark at 26 °C, respectively. For temperature treatments, the rice seedlings were grown in the growth chamber at constant temperatures of 30 °C and 22 °C, respectively, under 12 h of low light (LL, 80 µmol m^−2^ s^−1^)/12 h of dark.

### 4.2. Paraffin Sectioning and Transmission Electron Microscopy Analyses

For paraffin sectioning, the second leaf blades of the *sprl1* mutant and wild type at the two-leaf stage were harvested and fixed with a 70% formalin acetic acid–alcohol solution and then dehydrated in a gradient ethanol series. Subsequently, the samples were embedded in paraffin and cut into 8 μm-thick sections. After that, the sections were deparaffinized with xylene and washed in serial dilutions of ethanol. Finally, they were stained with safranin, destained with ethanol, stained with fast green, and observed with a microscope [36]. For the transmission electron microscopy, the full expanding leaves were harvested from the *sprl1* mutant and its wild type at the seedling stage. Then, the leaves were treated as follows: fixed in 3% glutaraldehyde, further fixed in 1% osmium tetroxide, dehydrated, embedded, sectioned, and stained. Finally, sections were observed under a JEM-1400FLASH transmission electron microscope (JEOL, Tokyo, Japan).

### 4.3. Histochemical Analysis

The fresh leaf blades of the *sprl1* mutant and its wild type were harvested from plants grown under normal conditions at the five-leaf stage. For the 3,3′-diaminobenzidin (DAB) or nitro blue tetrazolium (NBT) staining analysis, the samples were incubated in DAB or NBT staining solutions at 28 °C in the dark for 12 h, cleared in 95% ethanol, and then photographed [36]. For the trypan blue (TB) staining analysis, the samples were soaked in the TB staining solution for 10 min, heated over boiling water for 5 min and left to stain overnight, and finally destained with a chloral hydrate solution and photographed [51]. The contents of malondialdehyde (MDA) and the activities of catalase (CAT), superoxide dismutase (SOD), and peroxidase (POD) were measured using Assay Kits (#D799761, #D799597, #D799593, #D799591; Sangon Biotech, Shanghai, China).

### 4.4. Photosynthetic Pigment and Proto IX Quantification

For the pigment analysis, the leaves were collected from the *sprl1* mutant and its wild type at the seedling stage and the heading stage, respectively. The pigments were extracted from 0.2 g of fresh leaves with 80% acetone in the dark at 4 °C for 48 h. The contents of chlorophyll (Chl) and carotenoids (Caro) were measured at wavelengths of 470 nm, 646 nm, and 663 nm using the BIOMATE 3S UV-Visible Spectrophotometer (Thermo scientific, Waltham, MA, USA) and were calculated according to the method of Lichtenthaler and Wellburn [52]:

Chl *a* (mg/g) = ((12.21 × A663 − 2.81 × A646) × V)/(1000 × W);

Chl *b* (mg/g) = ((20.13 × A646 − 5.03 × A663) × V)/(1000 × W);

Caro (mg/g) = ((1000 × A470 − 3.27 × Chl a – 104 × Chl b) × V)/(1000 × W × 229)

For the Proto IX analysis, 0.1 g fresh leaves at the seedling stage were homogenized in 1 mL methanol/acetone/0.1 M NaOH (9:10:1, *v*/*v*/*v*), and the homogenates were centrifuged at 7197 g (Eppendorf 5430R; 7830 rpm) at 1 °C for 20 min. To oxidize the Protogen IX into Proto IX, 5 μL of 1 M acetic acid and 5μL of 2-butanone peroxide was added to 200 μL supernatant [28]. Then, the Proto IX was analyzed by HPLC on a C8 column (4.6 × 150 mm, 3.5μm; Waters) according to the method of Wang [53]. The elution profiles were detected by fluorescence, with excitation at 405 nm and emission at 625 nm [28]. The Proto IX was quantified by using the Proto IX standard (Frontier Scientific, Logan, UT, United States).

### 4.5. Vector Construction and Rice Transformation

For complementation of the *sprl1* phenotype, a 5528 bp genomic DNA fragment was amplified from the wild type 188R with the primer *OsPPO1*-Com F/R, digested with *Bam*HI, and then inserted into the pCAMBIA1300 vector using a one step cloning kit (Vazyme, Nanjing, China). The resulting plasmid pCAMBIA1300-*OsPPO1*, which contained a 1949 bp upstream sequence, an entire coding region, and a 333 bp downstream sequence of *OsPPO1*, was introduced into the *sprl1* mutant by Agrobacterium-mediated genetic transformation. The transgenic plants were detected using the primers *OsPPO1*-Tra F/R, locating on the *OsPPO1* gene and the pCAMBIA1300 vector, respectively (Table S1).

To over-express the *OsPPO1* gene, the full-length 1611 bp *OsPPO1* cDNA was amplified from the wild type 188R with the primers *OsPPO1*-OE F1/R1, digested with *Bam*HI/*Pst*I, inserted into the pCAMBIA2300-35S vector, and then introduced into the *japonica* variety Zhonghua 11 (ZH11). The transgenic plants were detected using the primers *OSPPO1*-OE F2/R2, locating on the *OsPPO1* gene and the pCAMBIA2300-35S vector, respectively (Table S1).

### 4.6. Subcellular Localization of the OsPPO1 Protein

The full-length cDNA sequence of *OsPPO1* was amplified from the wild type 188R with the primers *OsPPO1*-*GFP* F/R (Table S1), digested with *Sal*I, and inserted into the pCAMBIA2300-35S-eGFP vector. The pCAMBIA2300-35S-*OsPPO1*-eGFP and pCAMBIA2300-35S-eGFP vectors (negative control) were transformed into the 188R protoplasts and incubated overnight in the dark [54]. The green fluorescence signals were examined using a Leica EM CPD300 laser scanning confocal microscope.

### 4.7. Gene Expression Analysis

The total rice RNA was extracted from the roots, stems, leaves, sheaths, and young panicles with an RNA isolater kit (Vazyme), and the first-strand cDNA was reverse transcribed using a reverse transcription kit (Vazyme). The quantitative real time RT-PCR (qRT-PCR) analysis was conducted with a real-time PCR system (qTOWER^3^ G, analytikjena, Jena, Germany). The *Actin 1* gene was chosen as a reference gene. Three biological replicates were carried out in each experiment, and Student’s *t* test was used for the statistical analysis. All of the primers used in the qRT-PCR analysis are listed in Table S2 [55,56,57,58].

### 4.8. Herbicide Treatment

For the germination test, the seeds of OE2, OE15, and the control cultivar ZH11 were surface-sterilized, sown on a half-strength Murashige and Skoog (MS) solid medium containing different concentrations of technical-grade oxyfluorfen (J&K Scientific, Newark, NJ, USA) or acifluorfen (Ruisi reagent, Chengdu, China), and then grown in the growth chamber at 25 °C for 10 days under continuous light after dark incubation overnight [34]. For the seedling growth test, two- or three-week-old rice seedlings were sprayed with 125 μM of a commercial oxyfluorfen called Goal^®^ (Yifan Biotechnology, Wenzhou, China) or 600 μM acifluorfen in a paddy field at sunny-day-dusk. 

### 4.9. Statistical Analysis

Excel in Office 2016 was used to perform the statistical analysis. All of the experiments were repeated independently for three replicates, and the data were subjected to statistical analysis using Student’s *t*-test, with a *p*-value less than 0.05 or 0.01 considered significant.

## Data Availability

The data presented in this study are available in the article or supplementary material.

## Supplementary Materials

The following supporting information can be downloaded at: https://www.mdpi.com/article/10.3390/ijms23105781/s1.
