# Supplementary material for: Mutation of Protoporphyrinogen IX Oxidase Gene Causes Spotted and Rolled Leaf and Its Overexpression Generates Herbicide Resistance in Rice
Source: Int J Mol Sci. 2022 May 21;23(10):5781. doi: 10.3390/ijms23105781 (PMC9146718; doi:10.3390/ijms23105781)
Supplement: Supplementary file 1 [file ijms-23-05781-s001.zip › ijms-1741178-supplementary.pdf]

Supplementary material

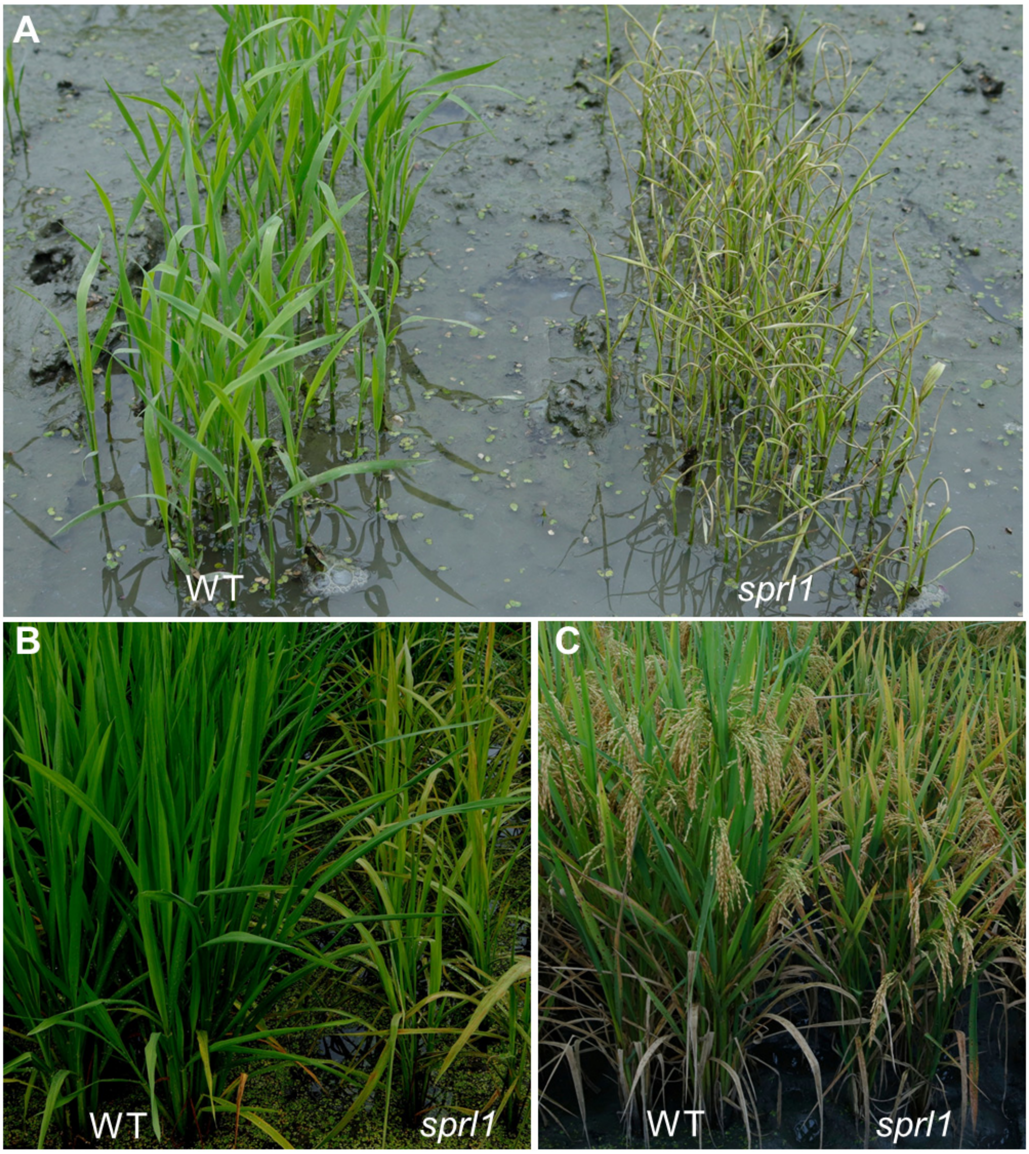

Figure S1. Plant phenotypes of the *sprl1* mutant and its wild type (WT). (**A**) Seedlings at the three-leaf stage. (**B**) Plants at the tillering stage. (**C**) Plants at the mature stage.

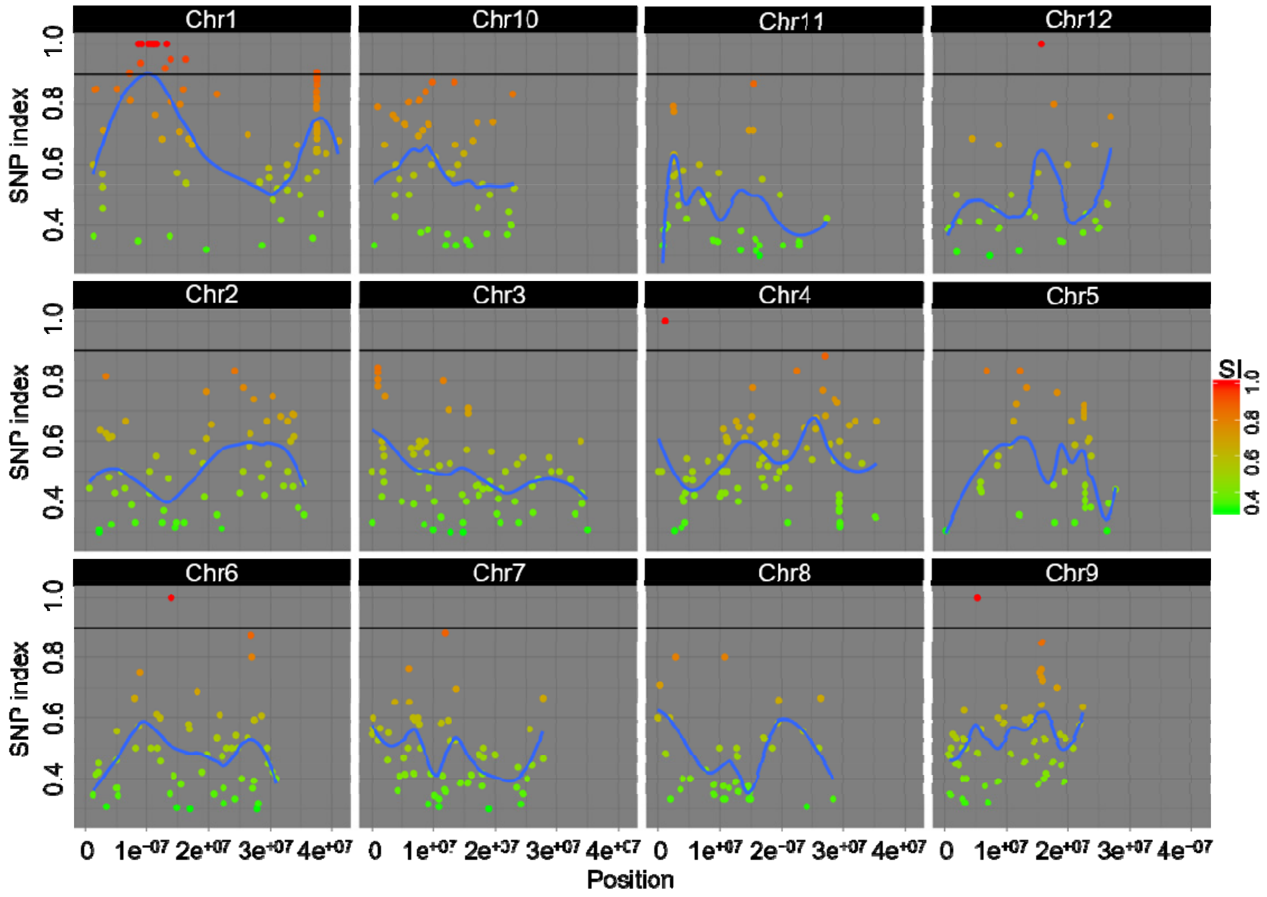

Figure S2. △(SNP-index) plot of the whole genome generated using MutMap.

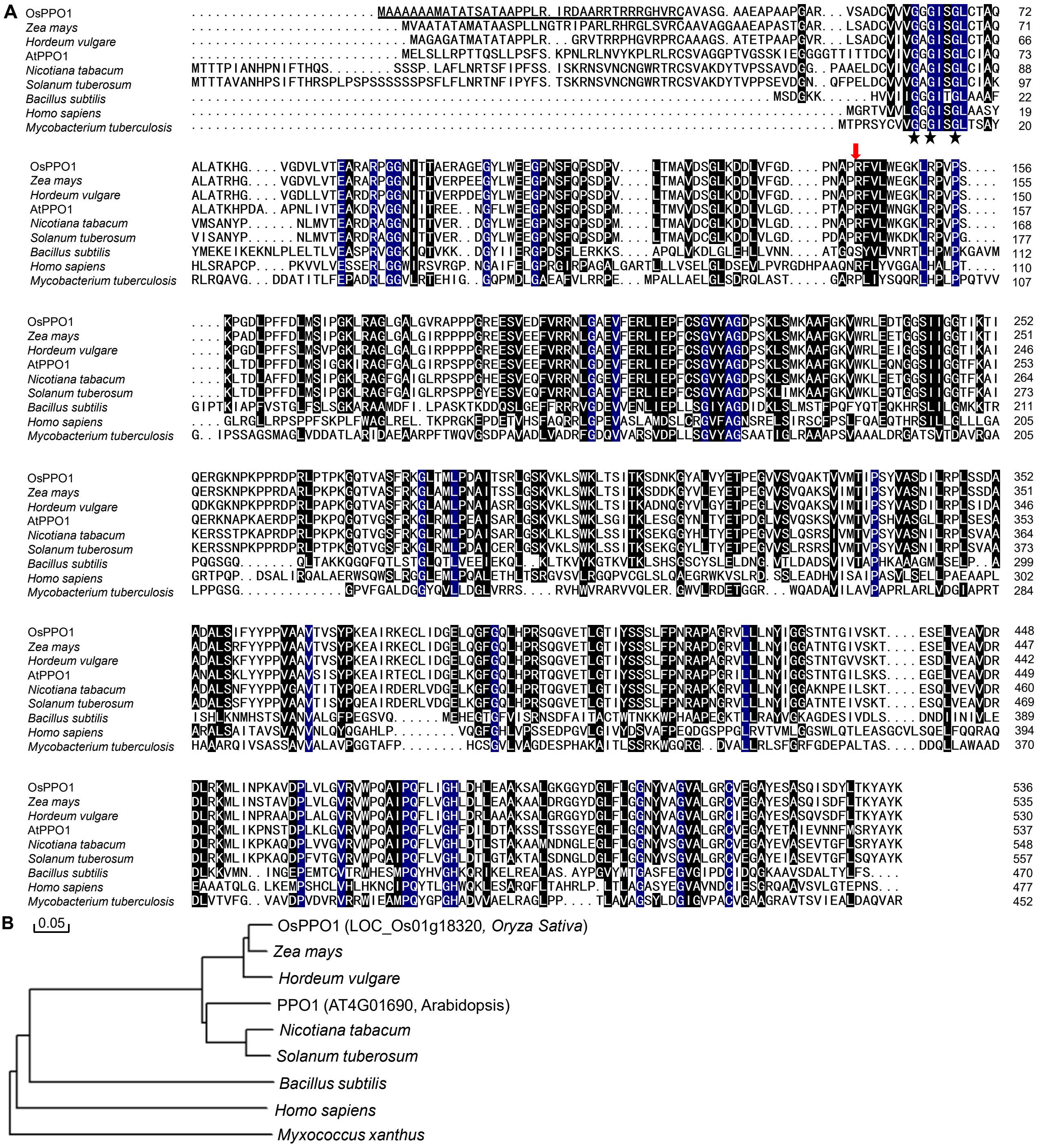

Figure S3. Sequence alignment and phylogenetic analysis of OsPPO1 and its homologues. (**A**) Sequence alignment. Identical residues are highlighted in blue, similar residues (≥75% identical) are highlighted in black. A black underline shows the putative chloroplast signal peptides. The red arrow indicates the mutant site of *sprl1*. The black asterisks shows the Gly-rich motif. (**B**) Phylogenetic tree representing alignment of *OsPPO1* homologs. The unrooted neighbor-joining tree using percentage identities was constructed based on a multiple sequence alignment produced with the program DNAMAN. Accession numbers for the respective sequences are as follows: rice (OsPPO1, LOC_Os01g18320), *Zea mays* (AAF26417.1), *Hordeum vulgare* (KAE8789512.1), Arabidopsis (PPO1, AT4G01690, BAA11820.1), *Nicotiana tabacum* (CAA73865.1), *Solanum tuberosum* (NP_001275224.1), *Bacillus subtilis* (AOT51497.1), *Homo sapiens* (BAA07538.1), *Myxococcus xanthus* (WP_003413871.1). Scale represents percentage substitution per site.

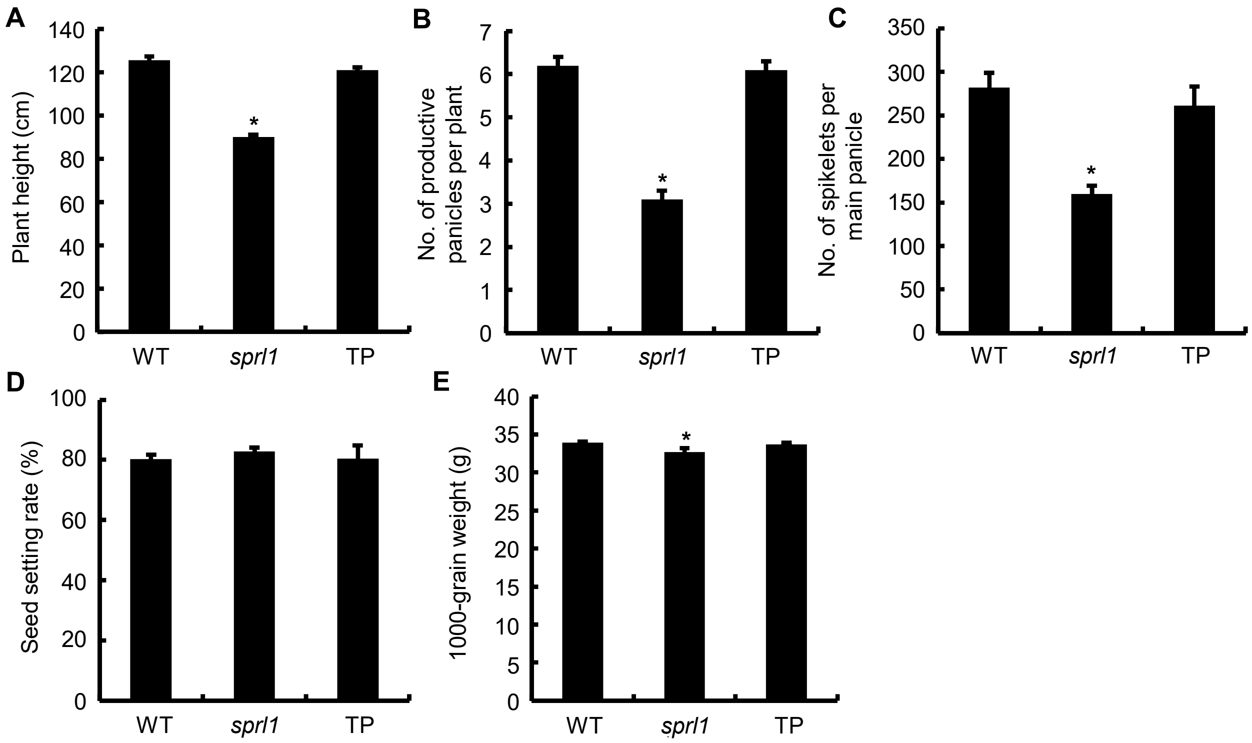

Figure S4. Major agronomic traits of complementation lines. Error bars represent standard errors of three independent experiments. The asterisk indicates statistically significant differences compared with the wild type at * $p < 0.05$. WT, wild type; TP, transgenic plants.

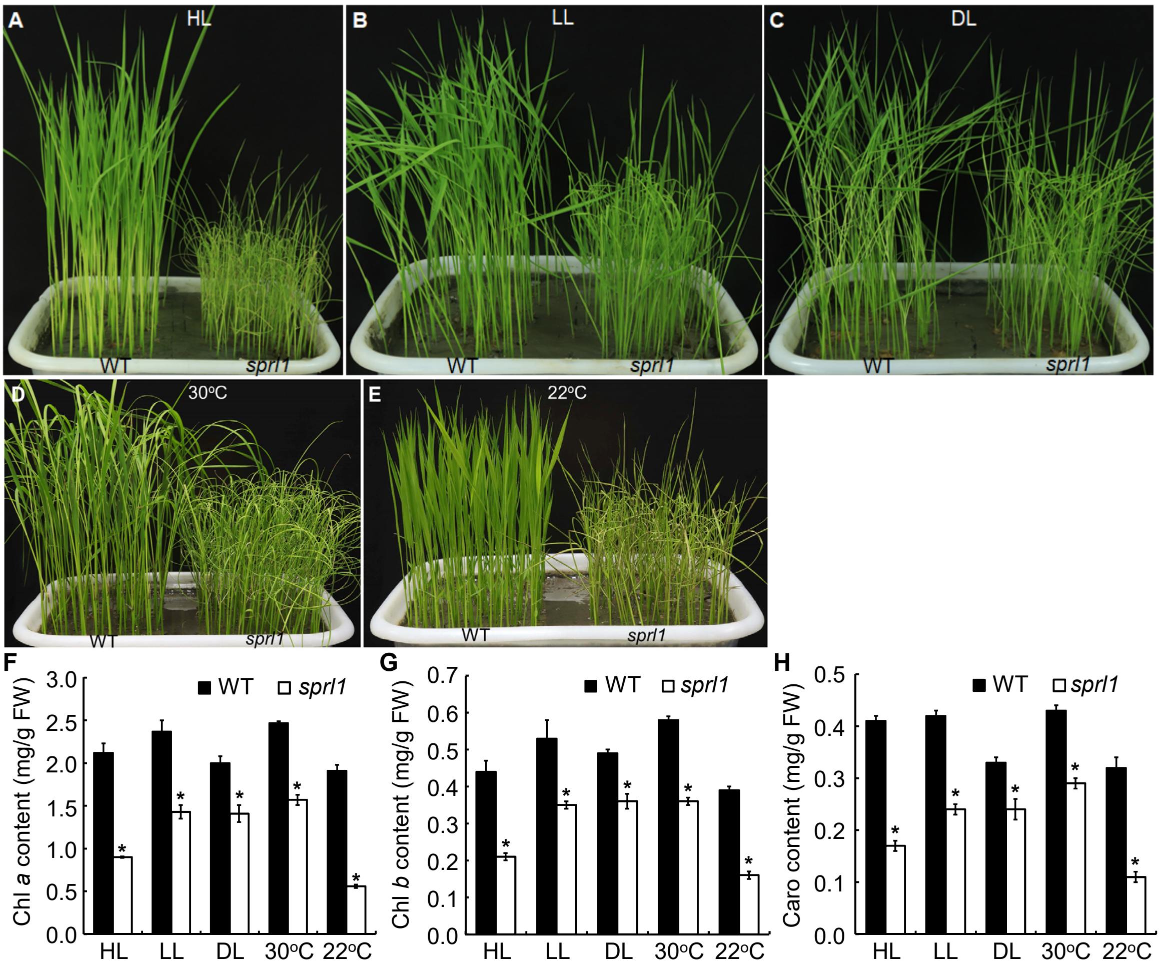

Figure S5. Phenotypic characterization of the *sprl1* mutant and WT grown in the growth chamber under various light intensity and temperature conditions. (**A**,**B**,**C**) 12-day-old seedlings grown under 12 h of high light (HL, 300 μmol m^{-2} s^{-1}), low light (LL, 80 μmol m^{-2} s^{-1}) and dim light (DL, 20 μmol m^{-2} s^{-1}) conditions at 28°C respectively/12 h of darkness at 26°C. (**D**,**E**) 12-day-old seedlings grown under constant 30°C and 22°C conditions respectively under 12 h of low light (LL, 80 μmol m^{-2} s^{-1})/12 h of darkness. (**F**,**G**,**H**) Contents of Chl *a*, Chl *b* and Caro respectively in WT and *sprl1* under various light intensity and temperature conditions. Data are means ± SD of three independent experiments. Asterisks indicate statistically significant between WT and *sprl1* mutant at * $p < 0.05$ by Student's *t* test.

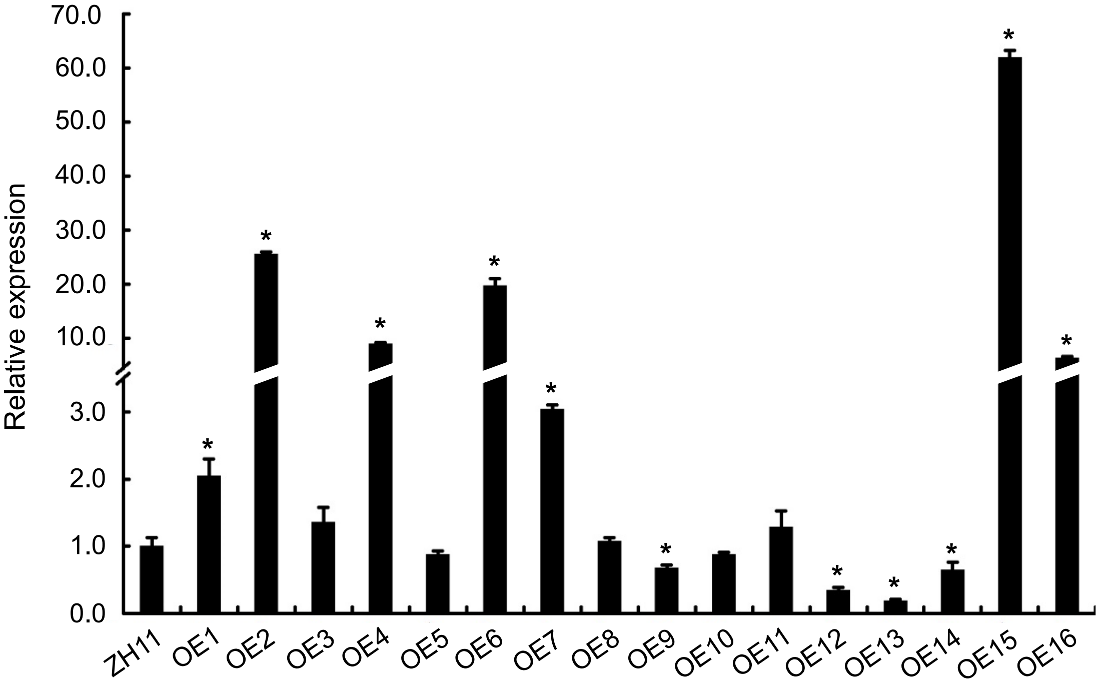

Figure S6. Relative expression levels of *OsPPO1* gene in the over-expressing lines. OE1-OE16 indicate transgenic lines overexpressing *OsPPO1* gene. The expression level in the control ZH11 was set to 1.0, and those in the over-expressing lines were calculated accordingly. Error bars represent standard errors of three independent biological replicates. Asterisk indicates statistically significant difference compared with the control ZH11 at $*p < 0.01$.

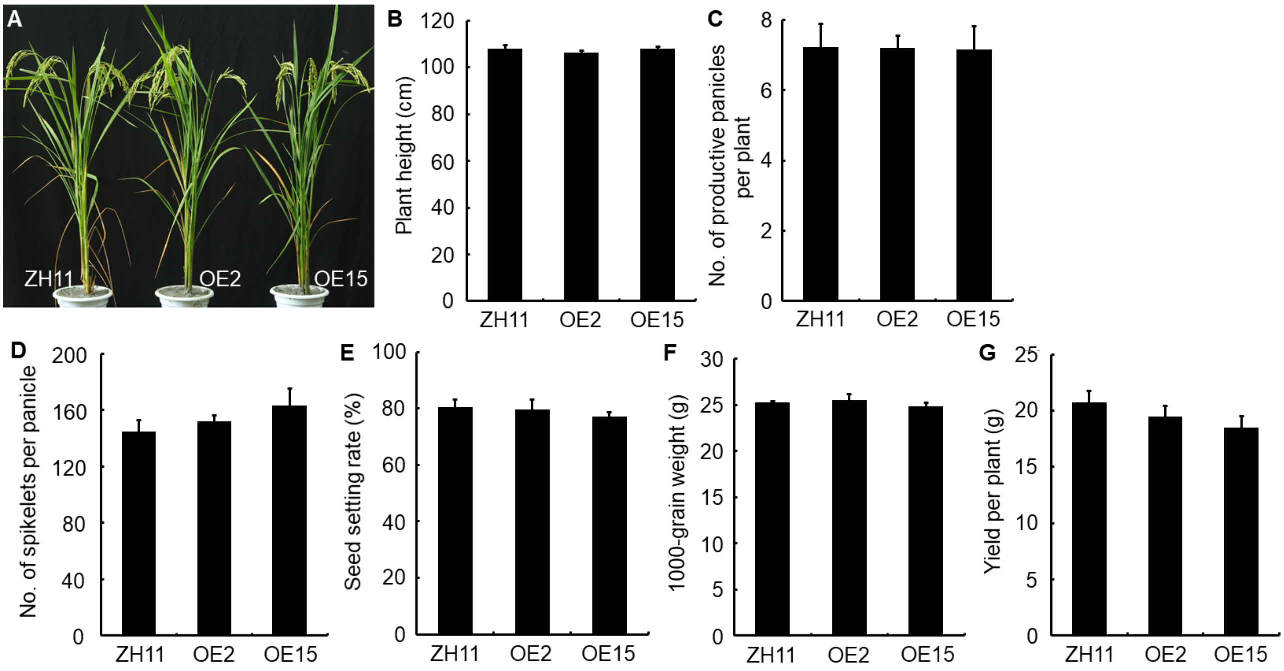

Figure S7. Comparison of major agronomic traits between the over-expressing lines and the control ZH11. Error bars represent standard errors of three independent experiments. The statistically significant differences were performed by Student's *t* test. $^{*}p < 0.05$.

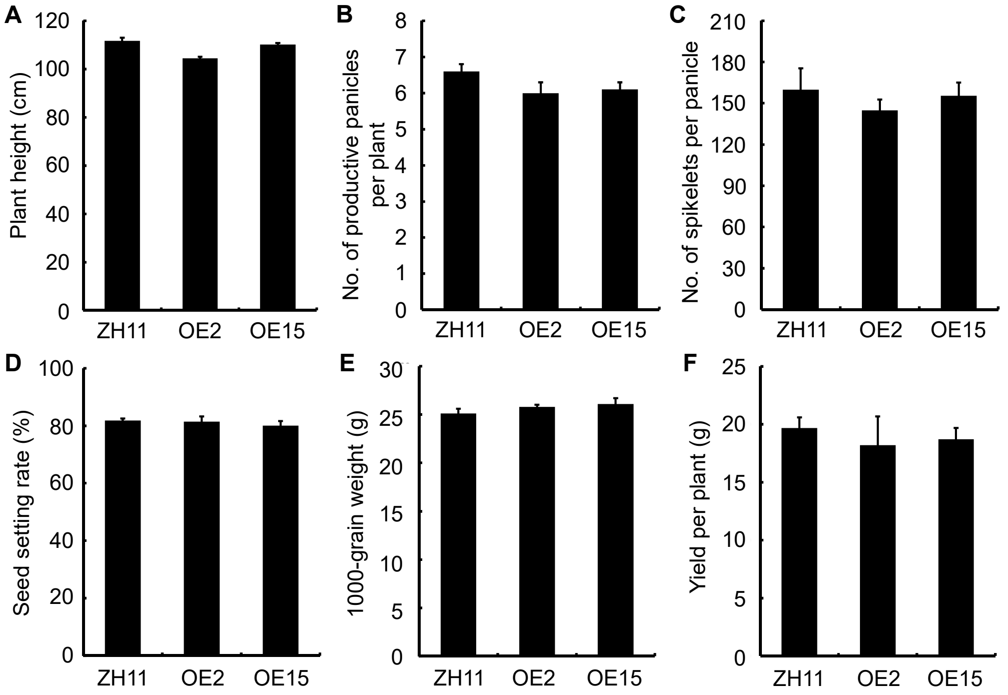

Figure S8. Comparison of major agronomic traits between the over-expressing plants with oxyfluorfen treatment and the control ZH11 without oxyfluorfen treatment. The 3-week-old seedling overexpressing *OsPPO1* gene were sprayed with 125 μM of oxyfluorfen, and wild-type ZH11 plants were sprayed with water as control. Error bars represent standard errors of three independent experiments. The statistically significant differences were performed by Student's *t* test. $^{*}p < 0.05$.

Table S1. Primers used in vector construction and detection

Marker	Forward primer (5′-3′)	Reverse primer (5′-3′)
OsPPO1-Com F/R	GAGCTCGGTACCCGGGGATCCAGGG	CAGGTCGACTCTAGAGGATCCGTCTGG
	CTGAGGCTGACATACGG	GAAGAGTCTAAACAG
OsPPO1-Tra F/R	TCGTATGTTGTGTGGAAT	CGTCCAATATGCTGCAGT
OsPPO1-OE F1/R1	GGACAGGGTACCCGGGGATCCATGG	AGAGCCCTGGCATGCCTGCAGTCACTT
	CCGCCGCCGCCGCAGCC	GTAGGCGTACTTGGT
OsPPO1-OE F2/R2	ATCCACCAGTTGCTGCTG	AATCAGTAAATTGAACGG
OsPPO1-Gfp F/R	CGGGGATCCTCTAGAGTCGACATGG	CACCATGGTACTAGTGTCGACCCACTT
	CCGCCGCCGCCGCAGCC	GTAGGCGTACTTGGT

Table S2. Primers used in qRT-PCR

Gene name	Forward primer (5′–3′)	Reverse primer (5′–3′)	Reference
OSPPO1	AGGTGTGTATGCTGGTGA	AGCATAGTCAGACCCTTC	
rbcL	CTTGGCAGCATTCCGAGTAA	ACAACGGGCTCGATGTGATA	Su et al., 2012
rbsS	CAGCAATGGCGGCAGGAT	AGGGCACCCACTTGGAACG	Li et al., 2015
psaA	GGAGGTGGCGAGTTAGTA	GATTTGCTTTATCGGGTAT	Li et al., 2015
psbA	TATGGGTCGTGAGTGGGA	TTATGCTCTGCCTGGAAT	Li et al., 2015
FC1	GGTCAACAGGGTGTAAAGAG	GTGCATCCAAGAGCTGGAAC	Inagaki et al., 2015
FC2	CTTGCCTTATGTTGGTGCTA	AGCCCCACTCCCATACAGTC	Inagaki et al., 2015
HEMA1	CGCTATTTCTGATGCTATGGGT	TCTTGGGTGATGATTGTTTGG	Su et al., 2012
GSAM	TGGACGTAAGGACATCAT	GTCCAAGTAATCGTAGGT	Wang et al., 2021
HEMB	ACCTGGATTGCCATACTT	TTCGTCGATCATGTTCAGTG	Wang et al., 2021
CHLD	CAAGGGTCGCCCAAGGTAAA	CTTCAGGTCCGAGAGTGCAG	Li et al., 2019
CHLH	CCAATCCGTAACCCGAAGGT	CAATAATTTTGGCGCTCTTCAA	Inagaki et al., 2015
CHLI	TTCGACAGGGATCCAAAGGC	ACAGCACCAAGGTTACTCCG	Li et al., 2019
DVR	CCATTGCCAGTTTCTTGGTG	AATTGAATGGCTAATGGCGT	Inagaki et al., 2015
PORA	TGTACTGGAGCTGGAACAACAA	GAGCACAGCAAAATCCTAGACG	Su et al., 2012
YGL	GGCACTGCTAGGACTCAC	CCCAAGACGAAGAACGGT	Li et al., 2019
CAO1	GATCCATACCCGATCGACAT	CGAGAGACATCCGGTAGAGC	Su et al., 2012
CatA	CAACCGCAACGTCGACAACTTCTT	TTCACCGGCAGCATCAGGTAGTTT	Yang et al., 2020
CatB	GCTTGCTTTCTGCCCAGCGATAAT	AAATAGTTTGGGCCAAGACGGTGC	Yang et al., 2020
APX1	AGGTGCCACAAGGAAAGATCTGGT	TCAGCAGGGCTTTGTCACTAGGAA	Yang et al., 2020
APX2	TGGGAAGATGCCACAAGGAGAGAT	TCCGCAGCATATTTCTCCACCAGT	Yang et al., 2020
POD1	ACGTCGGGGTCGCCAACAAC	CGAACTCGTCCACCGACGCC	Yang et al., 2016
AOX1a	CTTCGCATCGGACATCCATTA	TCCTCGGCAGTAGACAAACATC	Yang et al., 2020
AOX1b	CCTGCTCAGTTCATCACCATCA	GCATAAAACGGAGTGACAATAGC	Yang et al., 2020
ROC5	ACGAGCTCTTCTTCGTTGGG	GCAAAACACCACGGCGTAAA	
ZHD1	CAGTTCTGTGACGAGGTCGG	ATGCATTTGCTCCCGGTCAT	
REL1	GCACTCCAGCTTGGAACTCT	CTGAGTGCAGTGATCGGAGG	
REL2	TTGCCTTGTTGGGAGTTGGT	TGCACTTCACAGGACAAGCA	
SRL1	TCCTCATCTCCTGCCTCCTC	GAACCAAGGGGTTGGGAGAG	
Actin 1	TGTATGCCAGTGGTCGTACCA	CCAGCAAGGTCGAGACGAA	Li et al., 2015

Table S3. Segregation of F_2 populations from the crosses between *sprl1* and its wild-type parent 188R

Combination	No. of total plants	No. of normal plants	No. of mutant plants	Expected ratio	χ^2	$\chi^2_{0.05}$
sprl1×188R	453	345	108	3:1	0.27	3.84

Table S4. Insertion/deletion (InDel) markers used for mapping of the *sprl1* locus

Marker	Forward primer (5′-3′)	Reverse primer (5′-3′)
L1	CAGAAGCCGCTCTTACTC	AAACCCAAATTCCAACAC
L2	GATTTCATGGTAGGGTTT	TACAGCGAGTGAGGTCTT
L3	GTTGGAGATGGCATTAGA	AAGGTAGACGGCAGTGAG
L4	GGTGTACCTACAAGCCCA	TCTGCTCAGATCCTGCGA
L5	TGGTGAAAGGCACCTAGC	TTGCTCCCACACCTGTCG
